# The Dental Institutions of Learning

**Published:** 1883-10

**Authors:** 


THE DENTAL INSTITUTIONS OF LEARNING.—The regular or winter sessions of the various dental institutions will begin during the present month, October, and if the attendance so early in the session at the University of Maryland Dental Department is a criterion to judge by, the number of dental students throughout the country will be greatly in excess of last year.

The unprecedented success of the first session of the University of Maryland Dental Department, continues to even a greater degree at the beginning of the present session, as the number of Matriculates at this time of writing, is far in advance of last year at the same time, and gives very certain assurance of a much larger class.

The course of instruction pursued in this Dental Department gives general satisfaction, as evinced by the testimony of those who were in attendance during the session of 1882-83.

The Infirmary practice is very large and constantly increasing, the facilities for securing such a practice for actual manipulations by students, being unsurpassed. Already the supply exceeds the demand.

Students from all parts of the country, from Maine to Texas, Germany, Hungary, etc., are among the present matriculates.

The already large and well arranged Infirmary and Laboratory Building will require additions, which it is contemplated to erect at an early day, in the form of two wings. Such additions will also give a very imposing front on Greene Street and the most extensive Infirmary Hall ever erected, with an uninterrupted light from every side from at least thirty-five large windows, the present Infirmary having twenty-five.

Since last session, important improvements have been added to the Infirmary Hall, *every one* of the large number of dental chairs being now connected with a battery for the use of Electric Pluggers, together with an Electric Motor for the working of the Dental Engines.

